# Global Assessment of Genomic Regions Required for Growth in *Mycobacterium tuberculosis*

**DOI:** 10.1371/journal.ppat.1002946

**Published:** 2012-09-27

**Authors:** Yanjia J. Zhang, Thomas R. Ioerger, Curtis Huttenhower, Jarukit E. Long, Christopher M. Sassetti, James C. Sacchettini, Eric J. Rubin

**Affiliations:** 1 Department of Immunology and Infectious Diseases, Harvard School of Public Health, Boston, Massachusetts, United States of America; 2 Department of Computer Science, Texas A&M University, College Station, Texas, United States of America; 3 Department of Biostatistics, Harvard School of Public Health, Boston, Massachusetts, United States of America; 4 Department of Microbiology and Physiological Systems, University of Massachusetts Medical School, Worcester, Massachusetts, United States of America; 5 Department of Biochemistry and Biophysics, Texas A&M University, College Station, Texas, United States of America; McGill University, Canada

## Abstract

Identifying genomic elements required for viability is central to our understanding of the basic physiology of bacterial pathogens. Recently, the combination of high-density mutagenesis and deep sequencing has allowed for the identification of required and conditionally required genes in many bacteria. Genes, however, make up only a part of the complex genomes of important bacterial pathogens. Here, we use an unbiased analysis to comprehensively identify genomic regions, including genes, domains, and intergenic elements, required for the optimal growth of *Mycobacterium tuberculosis*, a major global health pathogen. We found that several proteins jointly contain both domains required for optimal growth and domains that are dispensable. In addition, many non-coding regions, including regulatory elements and non-coding RNAs, are critical for mycobacterial growth. Our analysis shows that the genetic requirements for growth are more complex than can be appreciated using gene-centric analysis.

## Author Summary

The significant rise in drug resistant strains of *Mycobacterium tuberculosis* has highlighted the need for new drug targets. Here, we present a novel method of defining genetic elements required for optimal growth, a key first step for identifying potential drug targets. Similar strategies in other bacterial pathogens have traditionally defined a set of essential protein-coding genes. Bacterial genomes, however, contain many other genetic elements, such as small RNAs and non-coding regulatory sequences. Protein-coding genes themselves also often encode more than one functional element, as in the case of multi-domain genes. Therefore, instead of assessing the quantitative requirement of whole genes, we parsed the genome into comprehensive sets of overlapping windows, unbiased by annotation, and scanned the entire genome for regions required for optimal growth. These required regions include whole genes, as expected; but we also discovered genes that contained both required and non-required domains, as well as non protein-coding RNAs required for optimal growth. By expanding our search for required genetic elements, we show that *Mycobacterium tuberculosis* has a complex genome and discover potential drug targets beyond the more limited set of essential genes.

## Introduction

Mutagenesis has long been a powerful tool for understanding the roles of genes and other chromosomal elements. Recently, high-density transposon insertion mutagenesis coupled with deep sequencing has enabled comprehensive identification of the required genes in many important bacterial pathogens [1]–[6]. Defining the protein-coding genes required for bacterial growth identifies both key biological processes and potential targets for drug development. However, protein-coding genes are not the only genetic elements that code for required functions. In densely packed bacterial genomes, many regulatory regions are required for appropriate expression of genes [7]. Moreover, all organisms produce large numbers of non-coding RNAs that can be important under a variety of growth conditions [8]–[10]. Gene-oriented analyses also look past cases wherein a single gene encodes several differentially important protein domains.

Here, rather than focusing on genes, we take an unbiased approach to create a comprehensive understanding of genomic requirement in *Mycobacterium tuberculosis* (Mtb). We model the Mtb genome as made up of “functional units”, a term that encompasses both genes and other genetic elements, many of which have yet to be annotated. By not limiting our analysis to whole-gene regions, we can find otherwise unidentified functional units while also gaining a more nuanced view of the genes required for mycobacterial growth, including critical domains within proteins and non-protein-coding regions that play important roles.

We find approximately 300 protein-coding genes wherein only portions of the coding sequence are required. These include genes, such as *ppm1* and *fhaA*, where we demonstrate that one domain is required for optimal growth whereas other domains are not. Our unbiased analysis also revealed required genomic elements in regions sitting between protein-coding genes. These include two RNAs, the tmRNA and the RNA component of RNaseP, which are required for optimal growth. In addition, we find a number of other regions that influence viability by uncharacterized mechanisms, but whose effects have previously been overlooked by gene-centric analyses.

## Results

### Deep sequencing for transposon insertion mapping

**Figure 1:**
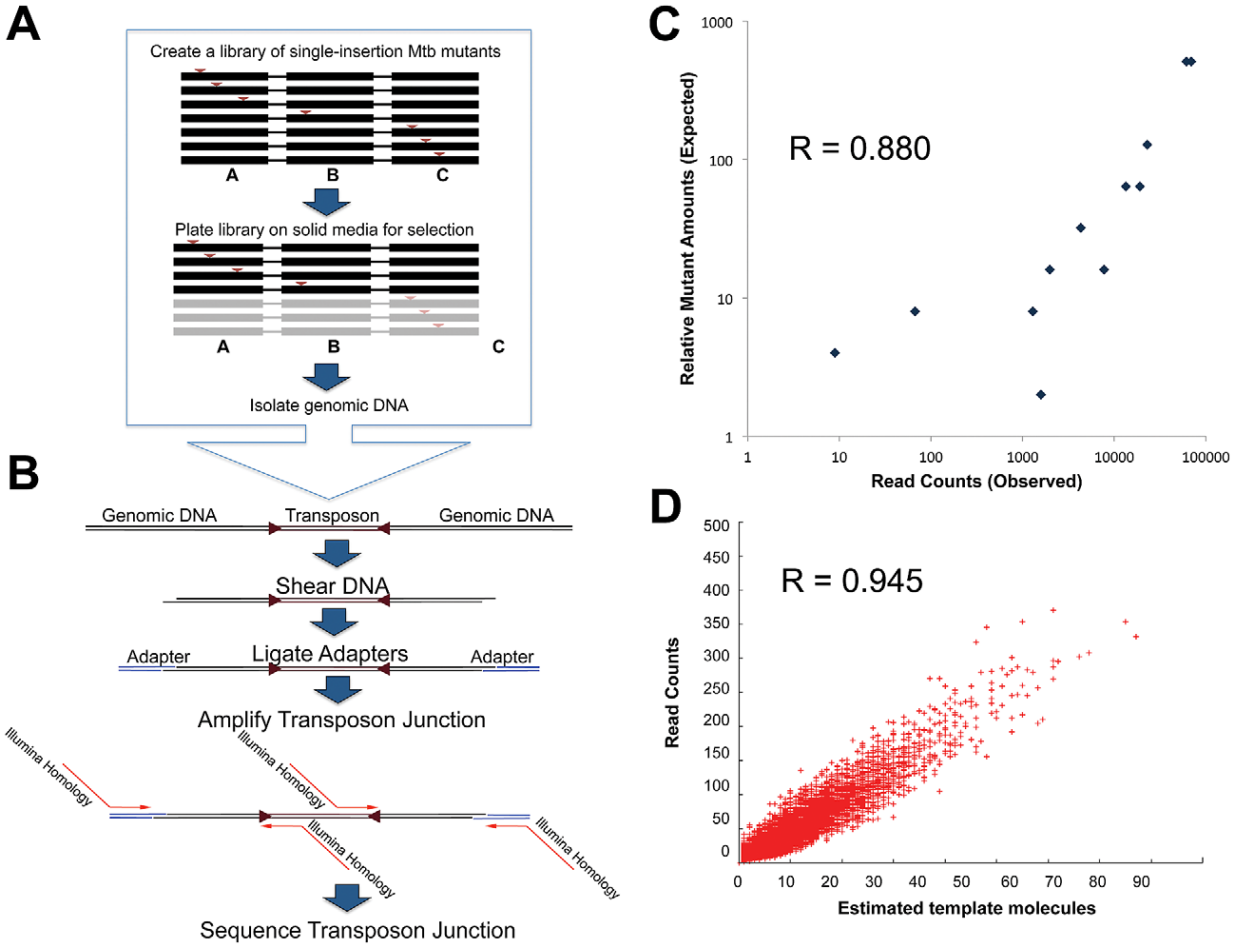
Transposon junction sequencing accurately reflects true library content. A. A Mtb mutant library is created by phage-delivery of transposons, disrupting each genome with a single insertion. Shown is a schematic of 6 mutant chromosomes spanning three genes (A–C), with transposons—red arrows—disrupting one of the three genes. After growing the library on 7H10 media, we pooled surviving mutants. In this schematic, gene C is required for optimal growth and thus mutants with transposons in gene C are lost. We isolated genomic DNA from the survivors for transposon site mapping. B. We sheared the genomic DNA by sonication, and repaired frayed ends to create blunt ends. We then used Taq polymerase to generate A-tails, allowing the ligation of T-tailed adapters. Finally, we selectively amplified transposon junctions using primers recognizing the transposon end and the adapter. Primers used for amplification contain all requisite sequences to permit direct sequencing of amplicons on an Illumina Genome Analyzer 2. C. We created a library of identified transposon insertion mutants in known relative quantities. DNA from the library was prepared for transposon junction sequencing. Insertion counts were plotted against the known relative quantity of the mutant in the library. D. To further confirm that read counts were a representation of the number of genomes in the library, we estimated the number of PCR template molecules. For each gene, we plotted the estimate of template molecule count against the read counts.

To perform a comprehensive assessment of Mtb's genetic requirements for growth, we used two ∼100,000-clone Mtb libraries generated through high-density transposon mutagenesis of the H37Rv strain [11]. We generated a library of single-insertion mutants by phage delivery of the Himar1 transposon, which randomly inserts into the genome at sites recognized by the TA dinucleotide (Figure 1A). We then created transposon-mapping probes by selectively amplifying and sequencing transposon-genome junctions using an Illumina Genome Analyzer 2. Using genome sequences adjacent to the transposon genomic sequences, we were able to map the insertion site of mutants in the library (Figure 1B) and count the reads mapped to each insertion site (insertion count, Table S1).

We reasoned that the insertion count should reflect the number of corresponding mutants in the library. To demonstrate this, we picked twelve individual transposon mutants and added each at a known quantity to a manually constructed library. Insertions were again mapped and counted by deep sequencing, and the insertion counts for each site was compared to the known relative quantities of each mutant in the pool (Figure 1C). Insertion counts were highly correlated with the known relative amount of each mutant (Pearson R = 0.880, p-value<0.0001, n = 14). Additionally, we confirmed that insertion counts accurately reflected the library's genome composition by counting the genome-transposon templates represented in our Illumina reads. Since random shearing events create the genome-transposon templates for amplification, the distance between the transposon and the sheared end represents a unique identifier for each template. We assessed the relationship between estimates of unique template molecules for each TA site and the read count for that site (Figure 1D), revealing excellent correlation (Pearson R = 0.945 p-value<0.0001 n = 36,488).

### Comprehensive map of genetic requirement in Mtb

In our Mtb library, transposon insertions occurred at 36,488 of the 72,927 possible insertion sites (TA dinucleotides). Each library generated an average of 2.3 million reads, resulting in a mean insertion count of 64 per hit-site. We counted the number of sequencing reads from each site in the two libraries and compiled the counts correcting for each library's total insertion count.

**Figure 2:**
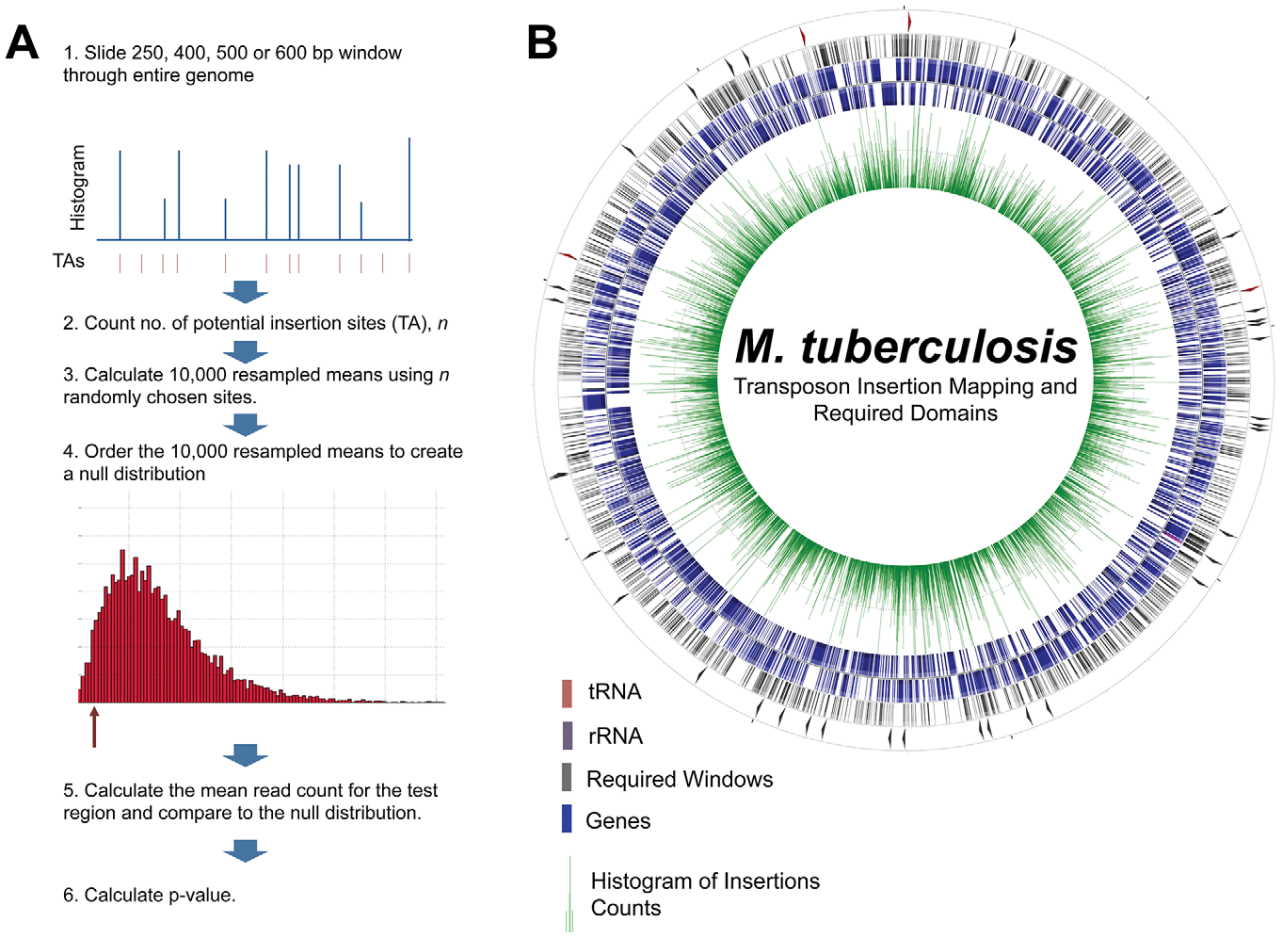
Functional requirement testing and mapping. A. Required regions were defined as regions with a statistical underrepresentation of insertion counts compared to the rest of the genome. To test this, we applied a non-parametric test for regions of increasing size, as described in B. Every 250, 400, 500, and 600 bp region (large enough for statistical power) was tested for insertion count underrepresentation, generating a comprehensive map of required regions in the Mtb genome. Tracks on the circularized genome, from inner-most to outer-most: 1. Histogram of insertion counts, 2. Annotated genes, forward direction, 3. Annotated genes, reverse direction, 4. All required regions. 5. Required intergenic regions.

Having demonstrated that insertion counts faithfully represent mutant numbers (Figure 1C), we used insertion counts to comprehensively assess the relative importance of selected genomic regions. We defined a region as required for optimal growth if total regional insertions were statistically underrepresented compared to genomic controls (Figure 2A). Required regions, therefore, are those in which mutations result in a statistically validated growth defect. We employed a non-parametric test to assess statistical underrepresentation and regions with a p-value of less than 0.01 and a false discovery rate (Benjamini-Hochberg) of less than 0.1 were defined as required for optimal growth *in vitro*.

Instead of assessing the requirement for growth of genomic regions based on predetermined gene coordinates, we divided the genome into contiguous overlapping windows to assess a comprehensive set of potential functional units. Our non-parametric test was powered to find significant regions containing at least 7 TA sites (6 or fewer precluded confident rejection of a null hypothesis of variation by chance alone). Thus, we focused on regions of sizes likely to contain 7 or more TA sites. The mean number of TA sites in windows of 400, 500, and 600 bp was 6.75, 8.45 and 10.12, respectively, and were thus used for our sliding window analysis of functional requirement for growth. Intergenic (IG) regions are relatively AT-rich in the Mtb genome, allowing us to add a 250 bp sliding window (mean of 6.20 TA sites in IG regions) to the analysis of IG regions. To lower the computational demands of this analysis, we chose to analyze every tenth window, reasoning also that functional units were unlikely to be smaller than 10 base pairs. Thus, we assessed the requirement for growth of every tenth 400, 500, and 600 bp window in the genome, along with every tenth 250 bp window in regions between protein-coding genes (Figure 2B, Table S4).

### Genes required for optimal growth

**Figure 3:**
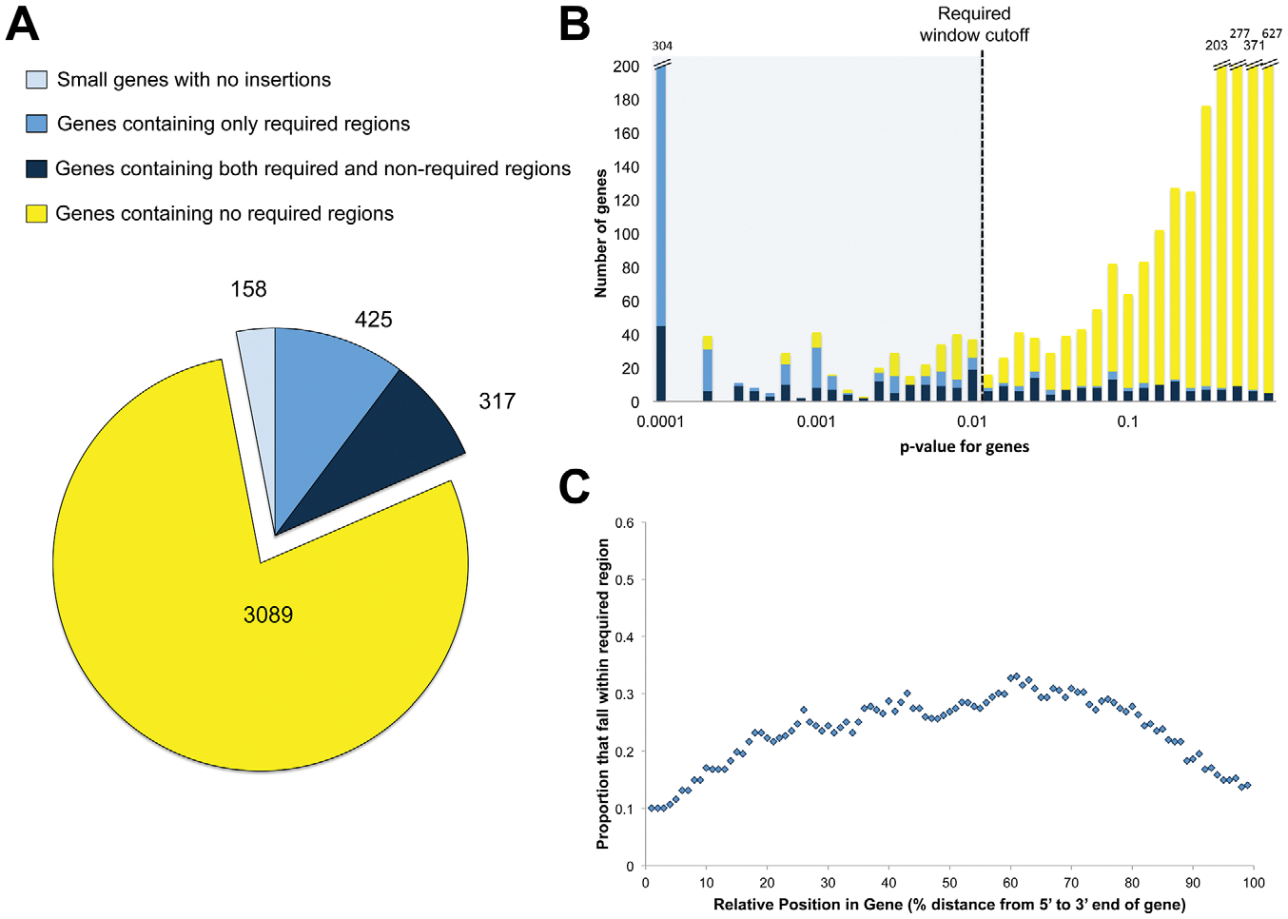
Domain discovery. A. Genes categorized by domain-level resolution of regional requirement. B. Genes categorized as containing only required regions (blue), containing both required and non-required regions (navy) and containing no required regions (yellow) were assessed for requirement along the entire length of the gene, creating a single p-value describing the statistical underrepresentation of insertion reads within the whole gene. For each category, the number of genes across the range of p-values are plotted. C. For genes with both required and non-required regions, the likelihood that the relative position within the gene resides in a required region.

We overlaid the coordinates of known genes on the generated results to find those that contained regions required for optimal growth. Of the 3,989 annotated genes, 742 contained required functional units, 3,089 contained no required functional units, while 158 did not sustain insertions but also did not contain enough TAs to meet statistical requirements (Figure 3A, Table S2). As a screen for genes with multiple functional units of varying requirement, we searched for genes that contained both required and non-required regions. A total of 317 genes met these criteria (Figure 3A, Table S2).

Our finding that many genes contain both required and non-required regions suggested that using only whole genes for analysis could misrepresent their importance for growth. Either the entire gene could appear required for optimal growth or the entire gene would be considered dispensable, leaving no room for the possibility that only a segment of the gene might be required. To determine how our results would compare to a gene-centric analysis, we calculated the requirement for growth of each gene by applying the non-parametric test to the gene as a whole. As expected, genes with required segments had a wide range of p-values when assessed using annotated boundaries instead of unbiased overlapping windows (Figure 3B, dark blue bars). A total of 170 out of the 317 (53.6%) had p-values above 0.01, demonstrating that our sliding window strategy accounted for a significant number of required functional units that would be ignored by gene-only strategies (Figure 3B).

A transposon insertion into the 5′ end of a gene will often block production of the encoded protein, either by attenuating transcription or disrupting ribosome binding sites and initiation codons. Thus, we expected that regions required for optimal growth would tend to be found at the 5′ ends of predicted genes. Surprisingly, a plot of the likelihood of discovering an required functional region as a function of the intragenic location revealed a symmetric curve, demonstrating that the required regions discovered have an equal likelihood of residing on either end of the gene (Figure 3C). We hypothesized that this may be because the transposon contains a promoter that can direct downstream transcription. To test this, we took two strains that contained transposon insertions and measured mRNA expression upstream and downstream of the transposon (Figure S2A). In both cases, expression upstream of the transposon did not significantly change, while downstream expression increased (Figure S2B). This is consistent with the observation that downstream genes can be transcriptionally activated by transposon insertions [12]. In addition, mycobacteria are able to use several initiation codons thus making it more likely that truncated but functional proteins can be produced from internal start sites.

While we were able to use this analysis to make many novel observations, we also found that our results supported previous findings. The majority of genes (63%) described as fully required for growth were similarly required in microarray-based studies using transposon site hybridization (TraSH) (Figure S1A, Table S2) [13]. The increased resolution from deep sequencing demonstrated that genes with fewer than 7 TAs resulting in an undersampling that prevented statistically confident requirement assessments (a separate category for genes with 6 or fewer TAs that did not contain insertions is noted in Figure 3A and Table S2). Since this was not known previously, we predicted that the microarray-determined set of required genes would be biased towards small genes. This proved to be true. In genes predicted to be required by TraSH but not in this study, the average number of TAs was 9.90 (Figure S1B). In contrast, the average number of TAs in fully required genes from this study was 19.84, a fair representation of the average of all genes assessed (19.47). In fact, of the genes only determined to be required in TraSH and not in this study, 43% had 7 or fewer TAs, accounting for much of the discordance between the two methods.

A more nuanced analysis of Mtb transposon insertion maps defined essential genes as those that contained “gaps,” any statistically significant runs of potential insertion sites lacking transposon insertions [3]. As expected, genes found in our sliding window analysis to have both required and non-required regions were more concordant with essential genes found by sequencing using this gap analysis than with microarray approaches or whole-gene analyses of insertion counts. Of genes described in our approach as fully required, 97.1% were described as “essential” by Griffin et al (Figure S1A), a remarkable level of agreement given the differences in growth media between the two studies. The increased concordance extended to genes containing both required and non-required regions. Griffin et al. described 151 of these genes as essential, while microarray methods only deemed 81 to be essential. The search for required regions within genes, a feature of both analyses, allowed for the discovery of these regions in longer genes, as evidenced by the increase in average number of TAs within these genes (Figure S1B).

### Identification of the required glycosyl transferase domain in Ppm1

**Figure 4:**
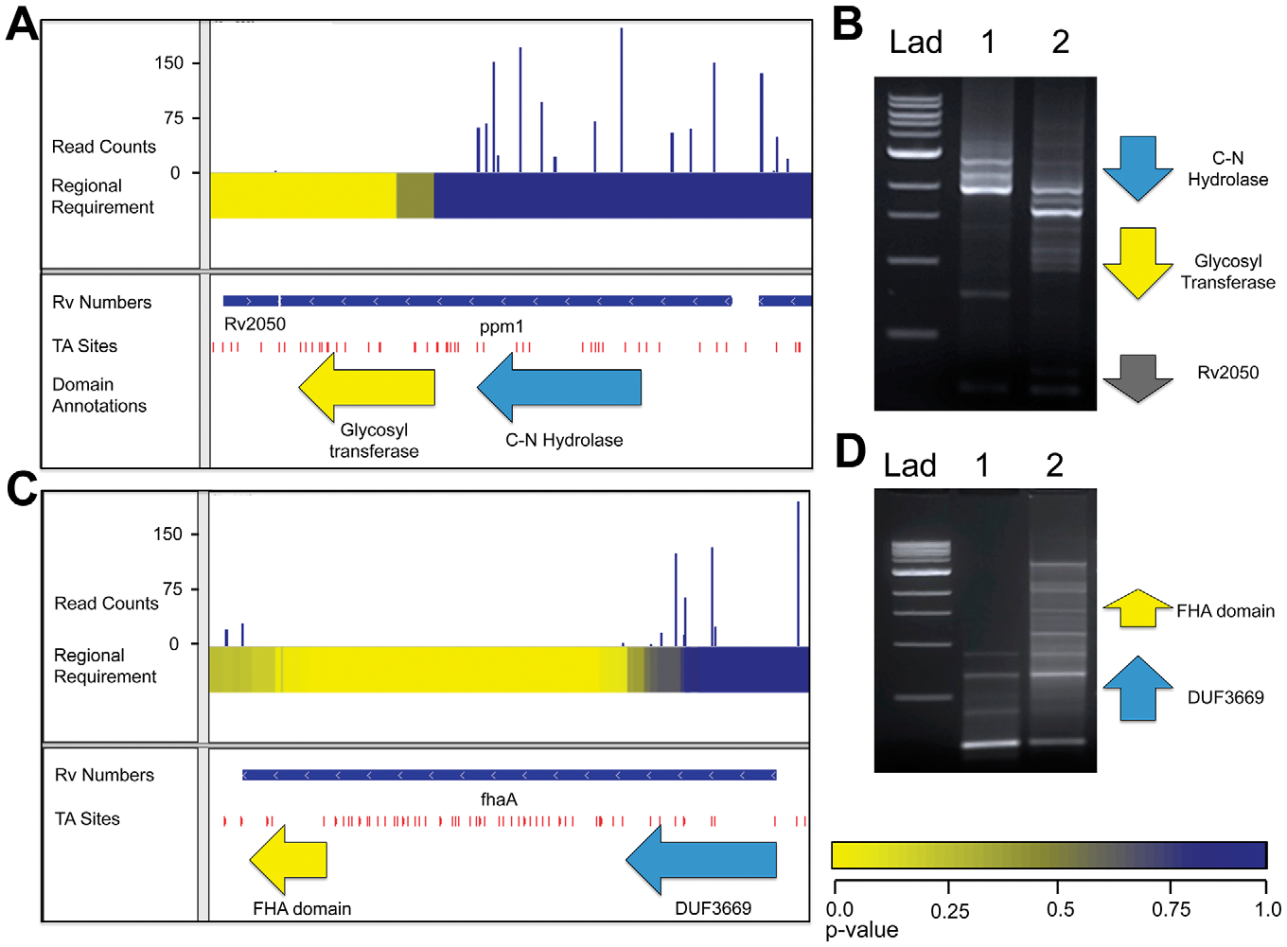
*ppm1* and *fhaA* each code for two domains with varying requirements for growth. A. IGV plot for genomic region containing *ppm1*. Tracks, from top to bottom: 1. Histogram of insertion counts, 2. Comprehensive heat-map of requirement of 500-bp windows, 3. Position of annotated genes, 4. TA sites, 5. Position of known domains within *ppm1*. B. PCR footprinting for insertions was performed using primers against the an upstream genomic region and the transposon end, resulting in amplicons spanning *ppm1* to various inserted transposons. Lad: 1 kb DNA ladder, 1: wt Mtb transposon library, 2: *ppm1*-complemented Mtb transposon library. C. IGV plot for genomic region containing *ppm1*. Tracks, from top to bottom: 1. Histogram of insertion counts, 2. Comprehensive heat-map of requirement of 500-bp windows, 3. Position of annotated genes, 4. TA sites. D. PCR footprinting for insertions was performed using primers against the an upstream genomic region and the transposon end, resulting in amplicons spanning *fhaA* to various inserted transposons. Lad: 1 kb DNA ladder, 1: wt Mtb transposon library, 2: *fhaA*-complemented Mtb transposon library.

We find that, in some genes, encoded domains have different effects on growth, accounting for the varying degrees of requirement across the gene's open reading frame. For example, the gene encoding Ppm1, an enzyme in the lipoarabinomannan (LAM) synthesis pathway, encodes a protein with two distinct domains. The region encoding the carbon-nitrogen hydrolase domain of Ppm1 sustained many insertions, while the region encoding the C-terminal glycosyl transferase was required for optimal growth (Figure 4A). While the specific requirement of the glycosyl transferase is a novel finding, it resonates with a previous report that only the glycosyl transferase was required for the synthesis of LAM, thought to be an essential cell wall component [14]. Another study revealed that Ppm1 has N-acyltransferase activity, which could be the non-required function of this two-domain protein [15].

To confirm that the lack of insertions in this domain was due to a functional requirement and not to insertional bias or the generation of toxic fusions or truncations, we created transposon libraries in the presence of a second copy of *ppm1*. We reasoned that a second copy would render the endogenous gene non-required and thus permissive for transposon insertion. We designed footprinting PCR primers upstream of the original *ppm1* to specifically generate amplicons containing transposon insertions into the endogenous copy (Figure 4B). Footprinting of the original library confirmed our sequencing results, as no insertions were found in the region encoding the glycosyl transferase. However, in the complemented library, that region did contain insertions, suggesting the glycosyl transferase is functionally required for growth. We further reasoned that only sense insertions—that is, insertions wherein the transposon's internal promoter is oriented in the same direction as the disrupted gene—would be tolerated in the 5′ end of *ppm1* to allow for the expression of the C-terminal required domain. To assess this, we used primers specifically designed to amplify sense and anti-sense insertions, and noted only sense insertions in the 5′ end (Figure S2C). In addition, we confirmed that many in-frame internal start sites exist between 5′ transposon insertion sites and the beginning of the 3′ domain.

### The MviN-binding domain of FhaA is required for growth

A recent report showed that FhaA was required for optimal growth of *Mycobacterium smegmatis* and postulated that the importance of the interaction of FhaA with the essential protein MviN for appropriate regulation of growth and peptidoglycan synthesis [16]. These processes are essential for mycobacterial cell division and cell wall biosynthesis. This work further demonstrated the C-terminal forkhead associated (FHA) domain of FhaA was required for MviN-binding, while an N-terminal domain of unknown function was dispensable for this interaction. In agreement with these findings, we show here that the region of *fhaA* encoding the FHA domain cannot sustain insertions, while the remainder of the gene is dispensable (Figure 4C). We used insertion footprinting to confirm these results, and found that the C-terminal insertion mutants were rescued for growth in the presence of a second copy of *fhaA* (Figure 4D).

Notably, both *ppm1* and *fhaA*, which we predict to be required for optimal growth based on the presence of a required region within these genes, were classified as non-essential in a previous microarray-based screen [13]. In fact, 247 of the 328 genes containing both required and non-required regions were not previously described as necessary for growth, likely due to the decreased spatial resolution of microarray-based methods. Microarrays limited the resolution of requirement testing to genes, and each gene received a single metric describing its requirement for growth. In addition, as our approach is not confined to gene boundaries, we have the additional resolution to identify domains within genes, as exemplified by *ppm1* and *fhaA*.

### The RNA components of RNaseP and the Clp quality control system are required for growth

**Figure 5:**
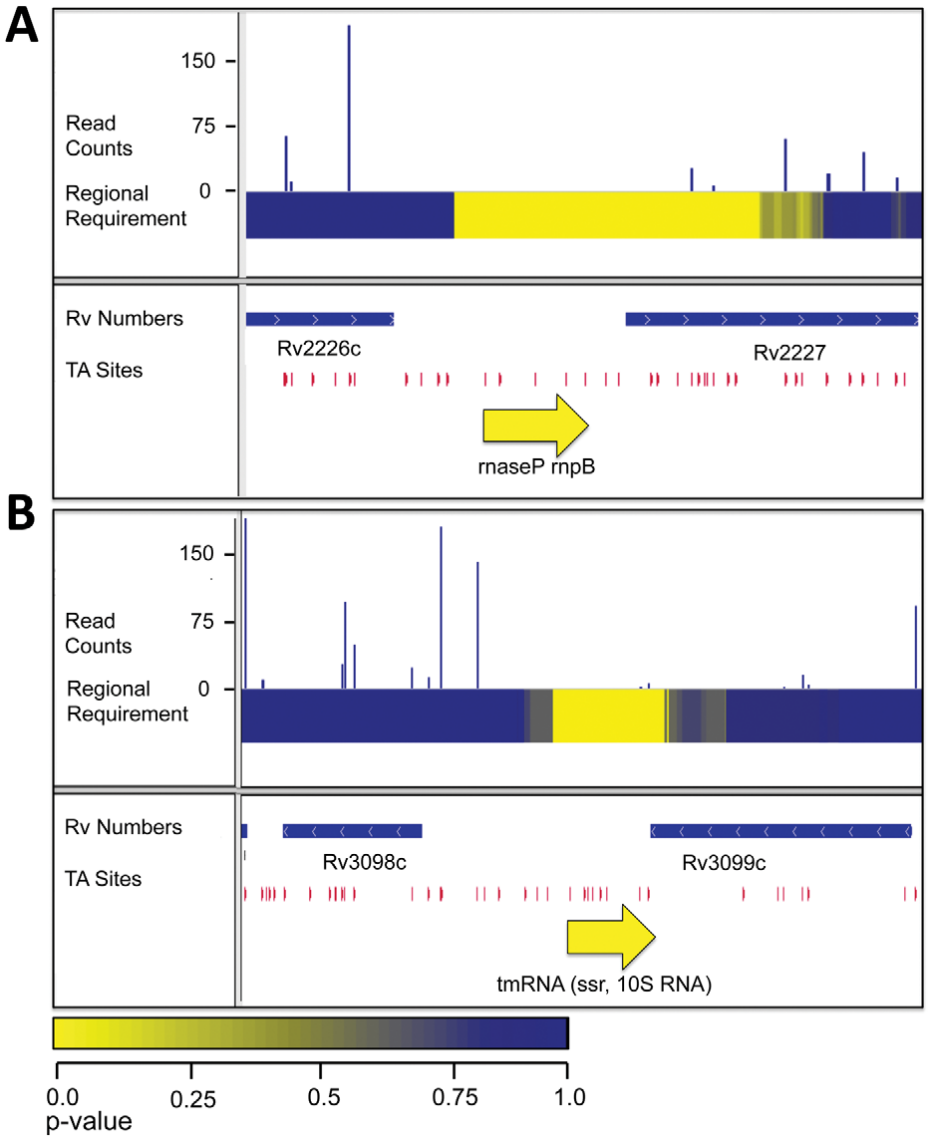
RNAs required for growth *in vitro*. A. IGV plot for genomic region containing the rnpB, the RNA component of RNaseL. Tracks, from top to bottom: 1. Histogram of insertion counts, 2. Comprehensive heat-map of requirement of 500-bp windows, 3. Position of annotated genes, 4. Position of TA dinucleotide sites, 5. Position of rnpB. B. IGV plot for genomic region containing the tmRNA. Tracks, from top to bottom: 1. Histogram of insertion counts, 2. Comprehensive heat-map of requirement of 500-bp windows, 3. Position of annotated genes, 4. Position of TA dinucleotide sites, 5. Position of the tmRNA.

Because we are not limited to annotated regions we were also able to probe the importance of intergenic regions. By scanning the genome for required 250, 400 and 500 bp regions, we found 25 intergenic regions required for optimal growth (Figure 2B and Table S3). These required intergenic regions contained many components of known essential cellular functions to be required for *in vitro* growth. These included 10 tRNAs as well as the RNA catalytic unit of RNaseP, which has been shown to be required for tRNA processing in other bacteria (Figure 5A). Additionally, one required intergenic region contained the tmRNA, a molecule required to release stalled ribosomes and to tag polypeptides for proteolytic degradation through an essential protease (Figure 5B) [17]–[18]. Of the intergenic segments containing functionally required regions, 11 had annotated functions and an additional 6 were adjacent to genes assessed as required for growth and, therefore, might contain promoters or other transcriptional regulatory elements. The remaining 19 required segments are situated between two non-required genes and, as yet, have no ascribed function.

## Discussion

Finding genetic loci that are required for optimal growth under specific conditions helps inform the basic understanding of bacterial physiology and efforts to develop new therapeutics for pathogens. Previously, we and others have used transposon mutagenesis to infer the requirement for genes under different growth conditions by utilizing the information provided by genome annotations [1]–[6]. Deep sequencing, which allows us to map precisely the insertion site of every mutant, affords a higher resolution assessment of genetic requirement, beyond just genes. Here, we demonstrate that an unbiased sliding window approach harnesses the full potential of this increased resolution. This approach identified not only whole genes required for optimal growth but also other required elements, such as non-protein coding RNAs and protein domains within insertion-containing genes, which would otherwise obscured by gene-centric analysis. An alternative analysis that uses significant gaps in insertion—rather than quantitative insertion counts—was also able to assess the requirement of protein domains (DeJesus et al., unpublished data, submitted). This analysis likely identifies regions absolutely essential for viability rather than all regions required for optimal growth.

We found that many genes contain elements that are important for growth even though other regions are not required. In at least two cases, *ppm1* and *fhaA*, published data have shown that the required regions encode specific protein domains. However, in other cases, these might represent non-protein-coding RNAs or cis regulatory elements. Bacteria encode many small RNAs many of which could be required for optimal growth and some of which are embedded within genes [8]–[10]. In addition, most genes have been annotated computationally, an uncertain pursuit that clearly can lead to misannotated start sites [19]. Genes with only 5′ insertions could fall into this category.

Similarly, important non-protein-coding regions could have multiple roles. In some cases, we found that known RNAs, such as *rnpB*, the catalytic RNA component of RNase P, and the tmRNA were required for optimal growth, supporting previous speculation [20]–[21]. Again, some other required regions might encode as yet unidentified non-coding RNA molecules. Still others might be promoters or other regulatory regions.

In this study, our resolution was limited by the specific properties of the *Himar1* transposon in mycobacteria. Our previous studies have shown that insertions are randomly distributed apart from the desired selection against insertion in essential regions [11], [22]. Despite this, we cannot assume that all sites lacking insertions represent required regions since unknown insertional biases of the transposon may exist. Thus, we defined a required region as one with a statistically underrepresented insertion count using a non-parametric test to account for such potentially unique biases within these data (Figure 2A). This allowed us to exclude, for example, windows with 6 or fewer TA sites, which demonstrably lacked power to distinguish a region as essential for growth relative to background variation. In GC-rich protein-coding regions, this limited our scope to windows of greater than 400 bp; less GC-rich intergenic regions allowed the assessment of windows greater than 250 bp. Thus, while we were able to identify many required protein domains and RNAs, it is certainly possible that smaller elements required for growth were missed due to these size constraints. This is a particular problem for non-coding RNAs that are often very small. For example, while we found 10 tRNAs required for growth, the remaining tRNAs reside in non-coding regions that did not have the requisite number of TA sites to determine requirement. Using the *Himar1* transposon in organisms with less of a GC bias, or in organisms in which a less restricted transposon exists, should result in increased resolution [4].

The analysis we used provides a powerful tool to perform functional genome analysis. Importantly, this type of approach is useful not only for single conditions, as we described but can also be used to identify elements critical under one growth condition but not another [23]–[25]. This is particularly important in organisms like Mtb, an obligate pathogen that never grows under conditions precisely comparable to those we use *in vitro*. Coupling high-density insertion libraries with deep sequencing and analytic methods such as that described here provides a powerful experimental tool for functional genome annotation.

## Methods and Materials

### Genomic library creation

Two independent libraries of 100,000 mutants were generated in the Mtb strain H37Rv as previously described on 7H10 agar [11]. Independent libraries were also generated in Mtb strains overexpressing *ppm1* and *fhaA*. Genomic DNA was isolated from each library and randomly fragmented to 400–600 bp pieces by sonication with a Covaris E220. Nicked ends were repaired (Epicentre end repaired kit), and A-tails were added with Taq polymerase to allow the ligation of T-tailed adapters. Transposon-junctions were amplified for 30 cycles (94 degrees, 30 seconds; 58 degrees, 30 seconds; 72 degrees, 30 seconds) using a primer recognizing the transposon end (5′-AATGATACGGCGACCACCGAGATCTACACTCTTTCCCTACACGACGCTCTTCCGATCCGGGGACTTATCAGCCAACC-3′) and one recognizing the adapter (5′-CAAGCAGAAGACGGCATACGAGATCGGTCTCGGCATTCCTGCTGAACCGCTCTTCCGATCGTCCAGTCTCGCAGATGATAAGG-3′). Primers used during amplification contained all the requisite sequence for binding to the Illumina sequencing platform. A 250–400 bp fragment of the amplicon was isolated from a gel and sequenced on an Illumina GA2 instrument with a custom sequencing primer (5′-TTCCGATCCGGGGACTTATCAGCCAACC-3′).

### Sequencing analysis

Reads from the Illumina sequencing run were first screened for the presence of sequence from the end of the Himar1 transposon. The following 35 bases were mapped to the Mtb genome, allowing for 2 mismatches. Reads that mapped to the genome at a TA site were designated as mapped insertions. Reads that mapped to multiple sites were randomly assigned to one of the mapped sites. For each library, the number of reads mapping to each site (insertion counts) was counted. Insertion counts were plotted on IGV and CGViewer [26]–[27].

### Requirement testing

For every possible region size containing *x* potential insertion sites, a null distribution of mean read counts was generated by calculating the mean read counts from a set of 10,000 randomly selected sets of *x* sites. The 10,000 randomly generated means were sorted and the rank of the test region's mean insertion count within the ordered null distribution was determined. The p-value was calculated as the rank of the test mean divided by the size the null distribution (10,000). Multiple test correction was performed by calculating the Benjamini-Hochberg false discovery rate over all regions tested. Regions containing 7 TA sites with no insertions had a p-value of 0.008 and an FDR of 0.06, while regions containing 6 TA sites with no insertions had a p-value of 0.018 and an FDR of 0.12. In order to power our study to detect required regions containing at least 7 TAs, we determined a region to be required for optimal growth if it had a p-value less than 0.01 and an FDR less than 0.1.

### DNA footprinting

Footprinting of transposon insertion sites was performed by PCR using a primer recognizing the Himar1 ITR sequence (5′-CCCGAAAAGTGCCACCTAAATTGTAAGCG-3′) and primers recognizing a genomic segment just upstream of the gene of interest. For directional footprinting, we used one primer to amplify sense insertions (5′-TTTTCTGGATTCATCGACTGTGGC-3′)—where the kanamycin resistance gene on the transposon was oriented in the same direction as the disrupted gene—and another for antisense insertions (5′-CAGCTCATTTTTTAACCAATAGGCCG-3′). Standard PCR conditions were used for long amplification with Phusion polymerase (94 degrees, 15 seconds; primer-dependent annealing temperature, 30 seconds; 72 degrees, 2 minutes).

## Supporting Information

Figure S1 A. For both fully required and partially required genes, the agreement with the essential gene set from Griffin et al. and with the required gene set from TraSH was calculated. B. Average length of genes (by number of TAs) are plotted for the following gene sets: required genes in TraSH, genes determined to be required in TraSH but not this analysis, fully required genes in this analysis, all genes assessed in this analysis, essential genes in Griffin et al., and all genes with required regions in this analysis.
(TIF)

Figure S2 A. Primer design for measuring expression upstream and downstream of the transposon. B. Relative expression of gene segments upstream and downstream of the transposon in transposon strains and H37Rv controls. C. Directional footprinting of transposon insertion. Lad: 1 kb DNA ladder, 1: wt *Mtb* transposon library, sense insertions, 2: *ppm1*-complemented Mtb transposon library, sense insertions, 3: wt *Mtb* transposon library, anti-sense insertions, 4: *ppm1*-complemented Mtb transposon library, anti-sense insertions.
(TIF)

Table S1 Read counts.
(TXT)

Table S2 Gene requirements.
(XLSX)

Table S3 Non-protein coding region requirements.
(XLSX)

Table S4 Sliding windows.
(ZIP)
