# Correction: Global Assessment of Genomic Regions Required for Growth in *Mycobacterium tuberculosis*

**DOI:** 10.1371/annotation/4669e9e7-fd12-4a01-be2a-617b956ec0bb

**Published:** 2013-07-25

**Authors:** Yanjia J. Zhang, Thomas R. Ioerger, Curtis Huttenhower, Jarukit E. Long, Christopher M. Sassetti, James C. Sacchettini, Eric J. Rubin

There was an error in the P-Values for this manuscript which affects tables 2 and 4, Figures 3 and S1 and the text of the Results section. The code used to analyze regional essentiality contained an error wherein the mean read count per TA site was rounded up to the nearest integer. A non-integer value was needed to generate a more accurate p-value using our non-parametric test. While this affected the p-value calculation for all regions, it affected the requirement call of only a small subset. Regions with fewer than 7 TA sites were determined to be non-required even if they had 0 reads, and so these regions were unaffected by the error. For regions of 7 TA sites or greater, correcting the rounding error changed the number of tolerated reads for a required region. It is worth noting that for larger TA sites, because more reads are tolerated, the integer approximation is an increasingly good estimate, so the rounding error mostly affected regions with 7 or 8 TA sites and 1-7 or 4-8 reads, respectively.

Specific changes:

Table 2: Gene essentiality calls. Calls and p-value summaries were changed to reflect analysis changes. Approximately 2% of gene calls were changed from non-required to containing required regions or vice versa. Please find the correct Table 2 here: www.plospathogens.org/corrections/ppat.1002946.t002.cn.xlsx

Table 4: Sliding window p-values. p-values were changed to reflect non-integer mean read calculations. Please find the correct Table 4 here: www.plospathogens.org/corrections/ppat.1002946.t004.cn.xlsx

Figure 3B: Histogram of requirement categories binned by whole-gene p-value estimates. The precise magnitude of each histogram bar is changed slightly, while the distribution remains the same. Relevant numbers are reflected in changes in the text, below. Please find the correct Figure 3 here: www.plospathogens.org/corrections/ppat.1002946.g003.cn.tif

Figure S1A: Overlap with previous studies. Our corrected analysis reveals somewhat improved overlap with both Sassetti et al. and Griffin et al. Changes in numbers are noted in textual changes, below.

Figure S1B: Average gene length of various requirement categories. The average length for genes with only required regions was changed from 19.84 to 19.42. The same change was reflected in the text, as noted below. Please find the correct Figure S1 here: www.plospathogens.org/corrections/ppat.1002946.s001.cn.tif

Changes to text in the Results section:

Old text: “Of the 3,989 annotated genes, 742 contained required functional units, 3,089 contained no required functional units, while 210 did not sustain insertions but also did not contain enough TAs to meet statistical requirements (Figure 3A, Table S2).”

New text: “Of the 3,989 annotated genes, 698 contained required functional units, 3,071 contained no required functional units, while 158 did not sustain insertions but also did not contain enough TAs to meet statistical requirements (Figure 3A, Table S2).”

Old text: “As a screen for genes with multiple functional units of varying requirement, we searched for genes that contained both required and non-required regions. A total of 317 genes met these criteria (Figure 3A, Table S2).”

New text: “As a screen for genes with multiple functional units of varying requirement, we searched for genes that contained both required and non-required regions. A total of 365 genes met these criteria (Figure 3A, Table S2).”

Old text: “A total of 170 out of the 317 (53.6%) had p-values above 0.01, demonstrating that our sliding window strategy accounted for a significant number of required functional units that would be ignored by gene-only strategies (Figure 3B).”

New text: “A total of 151 out of the 365 (41.3%) had p-values above 0.01, demonstrating that our sliding window strategy accounted for a significant number of required functional units that would be ignored by gene-only strategies (Figure 3B).”

Old text: “In contrast, the average number of TAs in fully required genes from this study was 19.84, a fair representation of the average of all genes assessed (19.47).”

New text: “In contrast, the average number of TAs in fully required genes from this study was 19.42, a fair representation of the average of all genes assessed (19.47).”

Old text: “Of genes described in our approach as fully required, 97.1% were described as “essential” by Griffin et al (Figure S1A), a remarkable level of agreement given the differences in growth media between the two studies. The increased concordance extended to genes containing both required and non-required regions. Griffin et al. described 151 of these genes as essential, while microarray methods only deemed 81 to be essential.”

New text: “Of genes described in our approach as fully required, 97.2% were described as “essential” by Griffin et al (Figure S1A), a remarkable level of agreement given the differences in growth media between the two studies. The increased concordance extended to genes containing both required and non-required regions. Griffin et al. described 185 of these genes as essential, while microarray methods only deemed 134 to be essential.”

Old text: “In fact, 247 of the 328 genes containing both required and non-required regions were not previously described as necessary for growth, likely due to the decreased spatial resolution of microarray-based methods.”

New text: “In fact, 231 of the 365 genes containing both required and non-required regions were not previously described as necessary for growth, likely due to the decreased spatial resolution of microarray-based methods.” 

