# Supplementary figures and images for: Global Assessment of Genomic Regions Required for Growth in Mycobacterium tuberculosis
Source: PLoS Pathog. 2012 Sep 27;8(9):e1002946. doi: 10.1371/journal.ppat.1002946 (PMC3460630; doi:10.1371/journal.ppat.1002946)

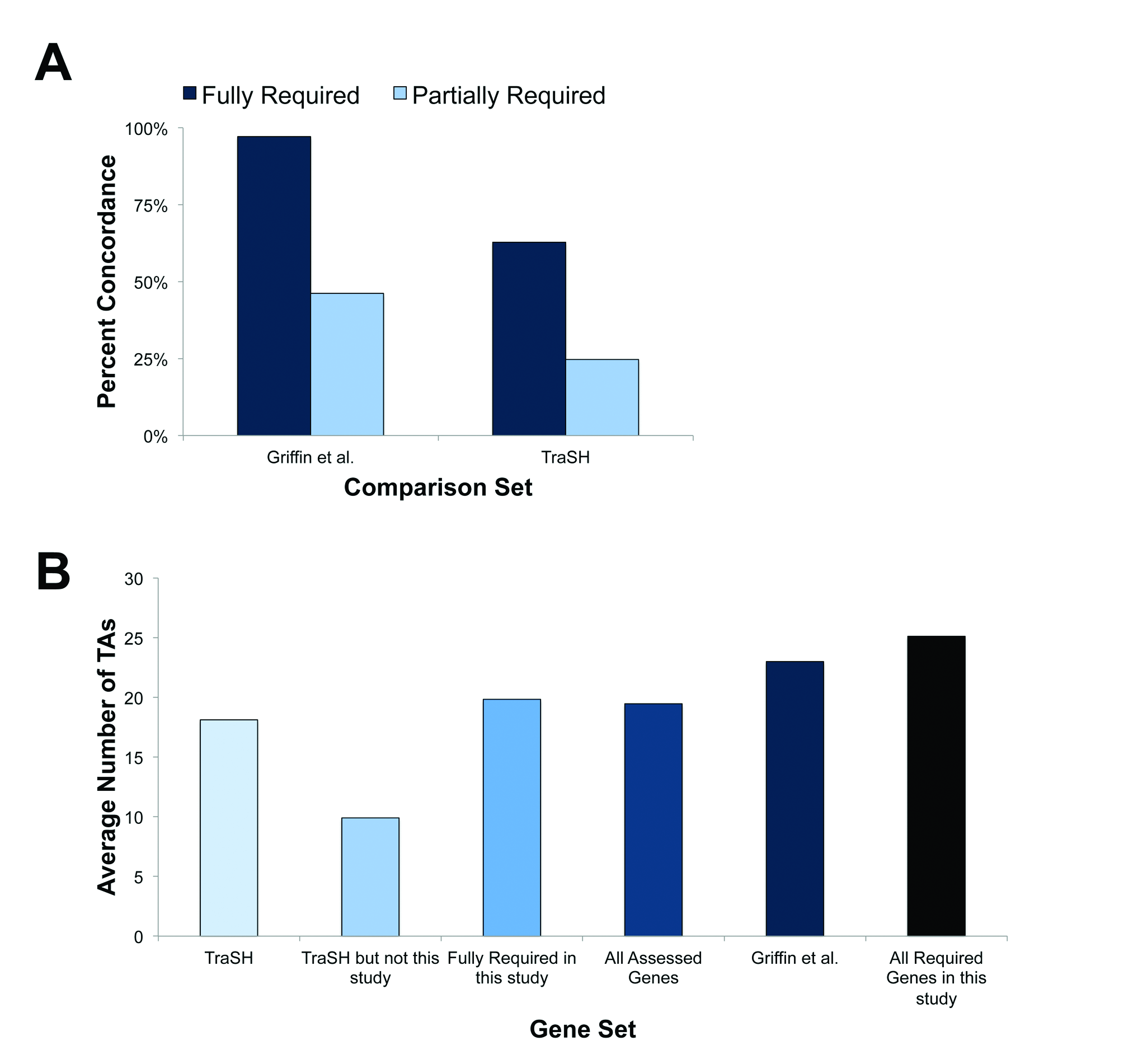

Supplement: Figure S1 — A. For both fully required and partially required genes, the agreement with the essential gene set from Griffin et al. and with the required gene set from TraSH was calculated. B. Average length of genes (by number of TAs) are plotted for the following gene sets: required genes in TraSH, genes determined to be required in TraSH but not this analysis, fully required genes in this analysis, all genes assessed in this analysis, essential genes in Griffin et al., and all genes with required regions in this analysis. (TIF) [file ppat.1002946.s001.tif]

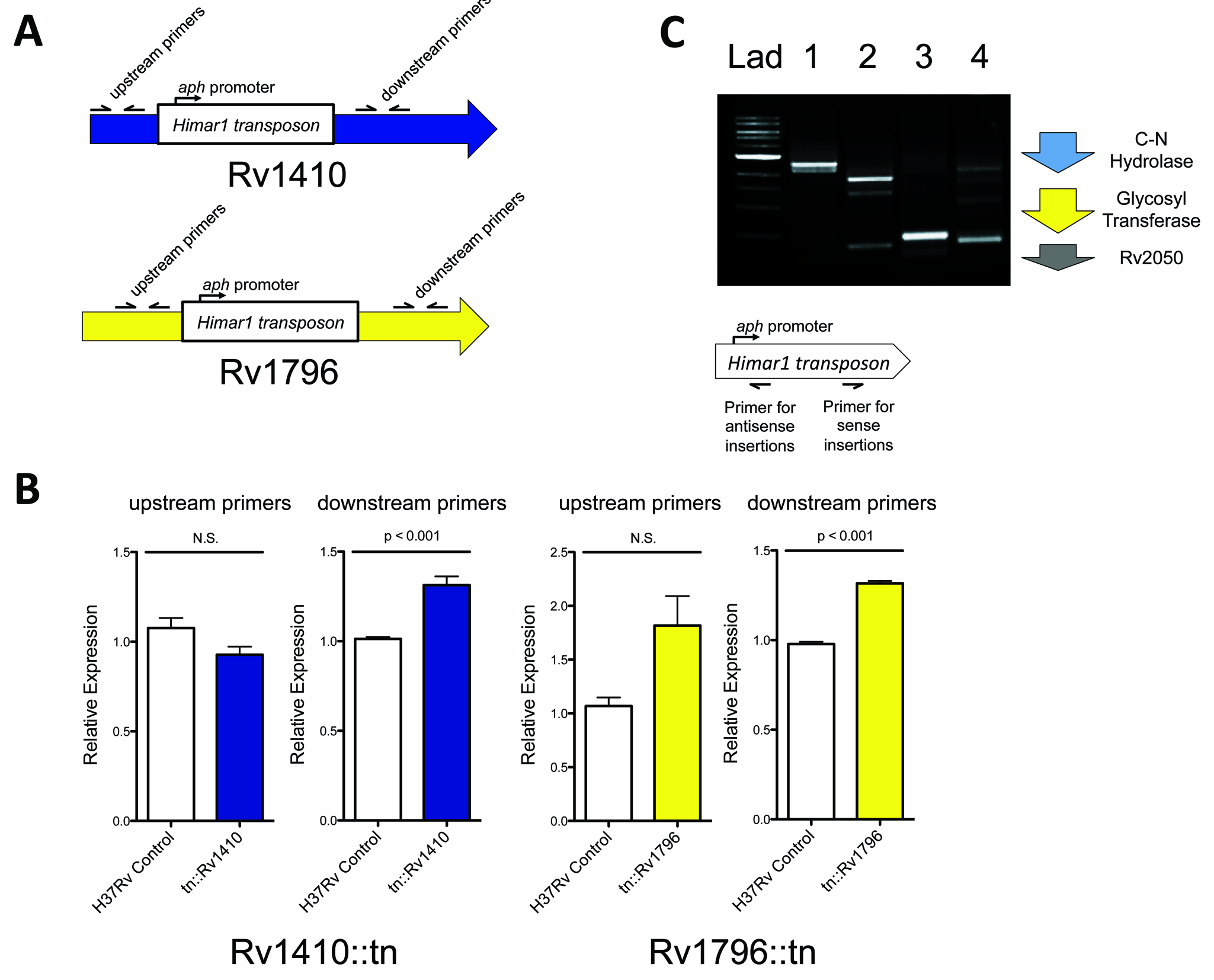

Supplement: Figure S2 — A. Primer design for measuring expression upstream and downstream of the transposon. B. Relative expression of gene segments upstream and downstream of the transposon in transposon strains and H37Rv controls. C. Directional footprinting of transposon insertion. Lad: 1 kb DNA ladder, 1: wt Mtb transposon library, sense insertions, 2: ppm1-complemented Mtb transposon library, sense insertions, 3: wt Mtb transposon library, anti-sense insertions, 4: ppm1-complemented Mtb transposon library, anti-sense insertions. (TIF) [file ppat.1002946.s002.tif]
